# Apolipoprotein E deficient rats generated via zinc-finger nucleases exhibit pronounced in-stent restenosis

**DOI:** 10.1038/s41598-019-54541-z

**Published:** 2019-12-03

**Authors:** Anne Cornelissen, Sakine Simsekyilmaz, Elisa Liehn, Mihaela Rusu, Nicole Schaaps, Mamdouh Afify, Roberta Florescu, Mohammad Almalla, Mauricio Borinski, Felix Vogt

**Affiliations:** University Hospital RWTH Aachen, Division of Cardiology, Pneumology, Angiology and Critical Care, Aachen, Germany

**Keywords:** Restenosis, Dyslipidaemias

## Abstract

The long-term success of coronary stent implantation is limited by in-stent restenosis (ISR). In spite of a broad variety of animal models available, an ideal high-throughput model of ISR has been lacking. Apolipoprotein E (*apoE*) deficient rats enable the evaluation of human-sized coronary stents while at the same time providing an atherogenic phenotype. Whereas *apoE* deficient rats have been proposed as animal model of atherosclerosis, to date it is unknown whether they also develop pronounced ISR. We sought to assess ISR after abdominal aorta stent implantation in *apoE* deficient rats. A total of 42 rats (16 wildtype, 13 homozygous *apoE*^−/−^ and 13 heterozygous *apoE*^+/−^ rats) underwent abdominal aorta stent implantation. After 28 days blood samples were analyzed to characterize lipid profiles. ISR was assessed by histomorphometric means. Homozygous *apoE*^−/−^ rats exhibited significantly higher total cholesterol and low-density cholesterol levels than wildtype *apoE*^+/+^ and heterozygous *apoE*^+/−^ rats. ISR was significantly pronounced in homozygous *apoE*^−/−^ rats as compared to wildtype *apoE*^+/+^ (p = <0.0001) and heterozygous *apoE*^+/−^ rats (p = 0.0102) on western diet. Abdominal aorta stenting of *apoE*^−/−^ rats is a reliable model to investigate ISR after stent implantation and thus can be used for the evaluation of novel stent concepts. Apolipoprotein E (*apoE*) deficient rats have been proposed as animal model of atherosclerosis. We investigated the development of restenosis 28 days after stent implantation into the abdominal aorta of wildtype *apoE*^+/+^, homozygous *apoE*^−/−^ and heterozygous *apoE*^+/−^ rats, respectively. Homozygous *apoE*^−/−^ rats exhibited significantly higher LDL and significantly lower HDL cholesterol levels compared to wildtype *apoE*^+/+^ and heterozygous *apoE*^+/−^ rats. Restenosis after stent implantation was significantly pronounced in western-diet-fed homozygous *apoE*^−/−^ rats, accompanied by a significantly increased neointimal thickness. Thus, *apoE* knockout rats exhibit elevated restenosis and might provide a novel tool for testing of innovative stent concepts.

## Introduction

Revascularization with percutaneous coronary intervention (PCI) and stent implantation is an effective therapy for reducing angina in stable ischemic heart disease and major adverse cardiac events in acute coronary syndromes^1^. In-stent restenosis (ISR), however, limits the long-term success of stents by the recurrence of symptoms and the necessity for a repeated revascularization at the treated site. We have learned important lessons on the underlying pathophysiology of ISR from both autopsy and animal studies^2,3^. PCI with angioplasty inevitably leads to endothelial denudation^4^, resulting in disturbance of the integrity of structures inside the diseased arterial wall^5,6^ and important alterations in the mechanical environment^7^. Within minutes, the arterial balloon injury site is covered by platelets and leukocytes^8,9^. The release of chemotactic factors and mitogens leads to the activation and proliferation of vascular smooth muscle cells (VSMCs) after 24–48 hours^10^. VSMC migrate from the media to the intima and shift from a contractile to a synthetic phenotype approximately 4–14 days after injury^11^. The VSMC proliferation and deposition of extracellular matrix proteins within 14 days to 3 months following PCI contribute to intimal thickening, neointimal hyperplasia and finally to the development of ISR^12,13^. Macrophages and lymphocytes persist at the stented vessel site for even more than 3 months^14^. Overall, human coronary arteries take approximately 6 months for complete healing after stent implantation.

A variety of animal models have been used to elucidate pathophysiological processes underlying ISR in order to direct research on its prevention. Each of these models features advantages and shortcomings, and an ideal animal model of ISR is lacking. As species have individual molecular and cellular arterial healing mechanisms, vascular lesions following stent implantation differ immensely across species. The porcine coronary artery model and the rabbit iliac artery model are the most frequently used animal models for ISR to date^15,16^. Swine and hypercholesterolemic rabbits have been demonstrated to develop lesions that are markedly similar in physiology and morphology to their human counterpart after stent implantation^17–19^. However, animal and housing costs are large in these models and especially the porcine model brings up logistical difficulties in long-term studies, including limitations on handling and equipment. Furthermore, only a limited number of antibodies to cellular proteins are available.

Murine models carry the advantages of high throughput, reproducibility and a broad availability of molecular markers as well as an ease of handling, housing and cost-effectiveness. Moreover, transgenic or knockout strains allow for the study of phenotypes of acquired or heritable metabolic disorders. As both rats and mice do not spontaneously form hemodynamically significant stenoses, the apolipoprotein E deficient mouse model has been broadly used to study cardiovascular diseases^20–22^.

The 299 amino acid glycoprotein apolipoprotein E (*apoE*) plays a significant role in cholesterol transport^23^. *ApoE* serves as a ligand for the uptake of chylomicron and very-low-density cholesterol (VLDL) remnants into hepatocytes by the LDL receptor related protein 1 and cell surface heparin sulphate proteoglycans^24^. Beyond its participation in plasma cholesterol lowering, *apoE* is known to have anti-inflammatory properties^25^. Deficiency in *apoE* leads to increased plasma levels of total cholesterol, mostly in the VLDL and chylomicron fractions^26^. Apolipoprotein E (*apoE*) knockout mice develop atherosclerotic plaques throughout the macrovasculature and are widely used for the investigation of atherosclerosis^27^. In response to stent injury, Ali *et al*. described significantly pronounced neointima formation in *apoE* deficient mice as compared to wild type animals^28^. The mechanism and time course of neointimal hyperplasia was reported to be similar to larger animals such as rabbits and pigs. Neointima was pronounced 28 days after stent deployment and consisted mainly of smooth muscle cells in a collagen and elastin rich matrix.

However, as it is not possible to implant commercially manufactured coronary stents into mice, stents have to be miniaturized involving important changes of the mechanic properties. Therefore, Langeveld *et al*. introduced the rat abdominal aorta stenting in 2004 using commercially available human coronary stents^29^. After Finn *et al*.^14^ and Indolfi *et al*.^30^ had reported a vascular healing course similar to larger animals after deployment of a small-caliber stent into the carotid artery of the rat, Langeveld *et al*. drew similar conclusions after aortic stent implantation^29^. A marked neointima was observed at 28 days after stent implantation, consisting of smooth muscle cells, extracellular matrix and few leukocytes around the stent struts. Oyamada *et al*. refined the technique of rat aorta stenting by introducing a trans-iliac access^31^. This resulted in a significantly higher survival rate as compared to trans-aorta implantation (89% vs. 43%). Wildtype rats, however, do not exhibit an atherogenic phenotype^15^. Recently, *apoE* knockout rats have been generated through nuclease techniques, such as Zinc Finger (ZF)^32^, Transcription Activator-Like Effector Nuclease (TALEN) technology^33^, or Clustered Regularly Interspaced Short Palindromic Repeats (CRISPR/Cas9)^34^. To date, it is unknown whether ISR is also pronounced in *apoE* knockout rats. Thus, we sought to characterize and quantify the impact of *apoE* deficiency in rats after the implantation of commercially available coronary stents into the abdominal aorta upon ISR development.

In order to assess ISR without the influence of antiproliferative coatings of drug-eluting stents, we decided to exclusively implant bare metal stents (BMS) and used a commercially available thin strut cobalt-chromium stent that has been well investigated in different multiple studies as well as randomized controlled trials^35^.

## Results

**Figure 1:**
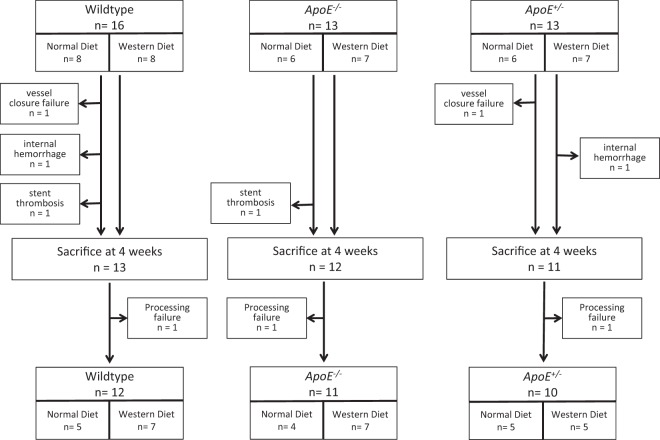
Study Design. Perioperative mortality was similar in wildtype *apoE*^+/+^ rats, homozygous *apoE*^−/−^ and heterozygous *apoE*^+/−^ deficient rats.

12 wildtype *apoE*^+/+^ (5 on normal diet, 7 on western diet), 11 homozygous *apoE*^−/−^ (4 on normal diet and 7 on western diet) and 10 heterozygous *apoE*^+/−^ rats (5 on normal diet and 5 on western diet) were analyzed for in-stent restenosis. Genotypes were confirmed via PCR using a specific primer for *apoE* (Supplemental Figs. 1–5). Figure 1 summarizes the study protocol. Prior to sacrifice, we assessed body weight and blood parameters of each animal.

### Characterization of body weight and blood parameter analysis

**Figure 2:**
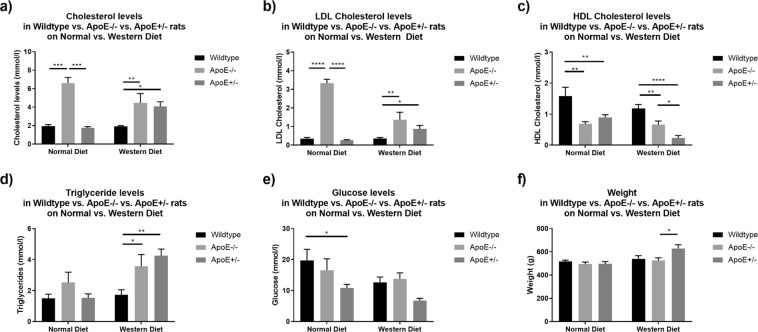
Lipid Profile, Glucose Levels, and Weight. Homozygous *apoE*^−/−^ rats exhibited significantly higher levels of total cholesterol (**a**) and LDL cholesterol (**b**) as compared to wildtype *apoE*^+/+^ and heterozygous *apoE*^+/−^ rats both on normal diet and on western diet. Whereas western diet feeding did not further increase total and LDL cholesterol levels in *apoE*^−/−^ rats, heterozygous *apoE*^+/−^ rats developed both elevated total and LDL cholesterol levels (**a**,**b**) as well as decreased HDL cholesterol levels (**c**) on western diet only. HDL cholesterol levels were significantly lower in both homozygous *apoE*^−/−^ and heterozygous *apoE*^+/−^ rats as compared to wildtype *apoE*^+/+^ rats. (**c**) Western diet led to increased triglyceride levels in homozygous *apoE*^−/−^ and heterozygous *apoE*^+/−^ rats. (**d**) Glucose levels (**e**) and weight (**f**) were generally similar in normal- and western-diet-fed rats. However, heterozygous *apoE*^+/−^ rats exhibited lower glucose levels than wildtype *apoE*^+/+^ rats on normal diet (**e**) and a higher body weight than *apoE*^−/−^ rats on western diet (**f**).

Body weight was in general similar in wildtype *apoE*^+/+^, homozygous *apoE*^−/−^ and heterozygous *apoE*^+/−^ rats (Table 1). In animals receiving western diet, however, body weight was higher than in animals on normal diet (503.21 ± 9.12 g vs. 549.28 ± 17.55 g; p = 0.0368). This was especially pronounced in heterozygous *apoE*^+/−^ rats (628.33 ± 32.91 g on western diet vs. 497.00 ± 19.04 g on normal diet; p = 0.0415, Fig. 2f).

**Table 1:** Body weight and blood parameter analysis.

	Wildtype *apoE*^+/+^ rats		Homozygous *apoE*^−/−^ knockout rats		Heterozygous *apoE*^+/−^ knockout rats	
	Normal Diet	Western Diet	Normal Diet	Western Diet	Normal Diet	Western Diet
Body weight (g)	530.1 ± 15.94		513.6 ± 16.45		546.3 ± 28.69	
	516.8 ± 11.47	539.57 ± 26.46	494.0 ± 17.78	524.86 ± 23.69	497.0 ± 19.04	628.3 ± 32.90
TC (mmol/l)	1.93 ± 0.08		5.1 ± 0.78		2.9 ± 0.46	
	1.94 ± 0.16	1.91 ± 0.07	6.6 ± 0.61	4.46 ± 1.01	1.74 ± 0.15	4.06 ± 0.52
LDL-C (mmol/l)	0.35 ± 0.04		2.1 ± 0.44		0.57 ± 0.14	
	0.35 ± 0.06	0.36 ± 0.06	3.33 ± 0.21	1.36 ± 0.41	0.26 ± 0.03	0.87 ± 0.18
HDL-C (mmol/l)	1.32 ± 0.13		0.67 ± 0.08		0.56 ± 0.12	
	1.52 ± 0.21	1.19 ± 0.13	0.68 ± 0.07	0.66 ± 0.12	0.89 ± 0.09	0.23 ± 0.08
TG (mmol/l)	1.6 ± 0.19		3.16 ± 0.50		2.97 ± 0.54	
	1.50 ± 0.28	1.67 ± 0.28	2.52 ± 0.67	3.44 ± 0.66	1.53 ± 0.26	4.40 ± 0.44
Glucose (mmol/l)	15.56 ± 2.02		14.61 ± 1.69		8.81 ± 0.94	
	19.68 ± 3.63	12.61 ± 1.77	16.53 ± 3.74	13.79 ± 1.93	10.86 ± 1.16	6.76 ± 0.72
Creatinine (μmol/l)	32.0 ± 1.85		31.09 ± 1.20		31.8 ± 1.26	
	34.4 ± 3.68	30.29 ± 1.76	34.5 ± 2.02	29.14 ± 0.94	33.80 ± 1.96	29.80 ± 1.16
Uric Acid (μmol/l)	46.92 ± 2.25		42.36 ± 3.13		49.6 ± 2.29	
	50.2 ± 3.35	44.57 ± 2.9	39.5 ± 3.57	44.0 ± 4.57	48.0 ± 3.65	51.2 ± 3.01
White Blood Cells (10^3^/μl)	10.81 ± 1.19		9.45 ± 1.03		9.32 ± 0.87	
	8.88 ± 0.18	12.19 ± 1.92	8.7 ± 1.41	9.87 ± 1.46	9.1 ± 1.78	9.54 ± 0.48

Data is presented as mean ± standard error of the mean.

Blood parameter analysis is summarized in Table 1. Total cholesterol (TC) and low-density lipoprotein cholesterol (LDL-C) levels were markedly elevated in homozygous *apoE*^−/−^ rats as compared to wildtype *apoE*^+/+^ and heterozygous *apoE*^+/−^ rats, both on normal chow and on western diet (Fig. 2a,b). Western diet did not lead to a further increase neither in TC nor in LDL-C levels in *apoE*^−/−^ rats with even higher LDL-C levels on normal-diet-fed *apoE*^−/−^ rats. Heterozygous *apoE*^+/−^ rats exhibited elevated TC and LDL-C levels on western diet only. In comparison with wildtype *apoE*^+/+^ rats, high-density lipoprotein cholesterol (HDL-C) levels were significantly lower both in homozygous *apoE*^−/−^ and in heterozygous *apoE*^+/−^ rats (Fig. 2c). Whereas similarly low HDL-C levels were observed both on normal and on western diet in homozygous *apoE*^−/−^ knockout rats, the lowering of HDL-C in heterozygous *apoE*^+/−^ rats was more pronounced on western diet (Fig. 2c). Both homozygous *apoE*^−/−^ and heterozygous *apoE*^+/−^ rats exhibited significantly increased triglyceride levels on western diet (Fig. 2d). Blood glucose levels were significantly lower in heterozygous *apoE*^+/−^ rats as compared to wildtype *apoE*^+/+^ rats on normal diet (10.86 ± 1.16 mmol/l vs. 19.68 ± 3.63 mmol/l; p = 0.0284). There were no differences in blood glucose levels between the other groups (Fig. 2e). Also, white blood cells, creatinine levels and uric acid levels were similar in wildtype *apoE*^+/+^, homozygous *apoE*^−/−^ and in heterozygous *apoE*^+/−^ rats irrespective of diet (Table 1).

### Histomorphometric measurements

**Figure 3:**
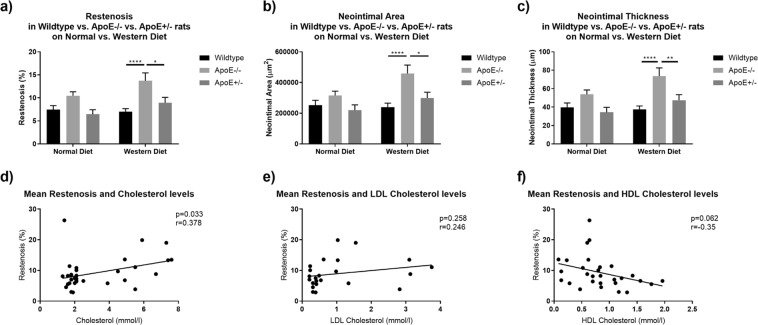
In-Stent Restenosis, Neointimal Area and Neointimal Thickness. Homozygous *apoE*^−/−^ rats exhibited pronounced restenosis, a significantly greater neointimal area and neointimal thickness on western diet. There were no significant differences in restenosis, neointimal area and neointimal thickness in rats on normal diet. (**a**–**c**) Irrespective of genotype, elevated total cholesterol levels correlated positively with ISR. (**d**) Neither LDL cholesterol nor HDL cholesterol levels were significantly associated with ISR (**e**,**f**).

**Figure 4:**
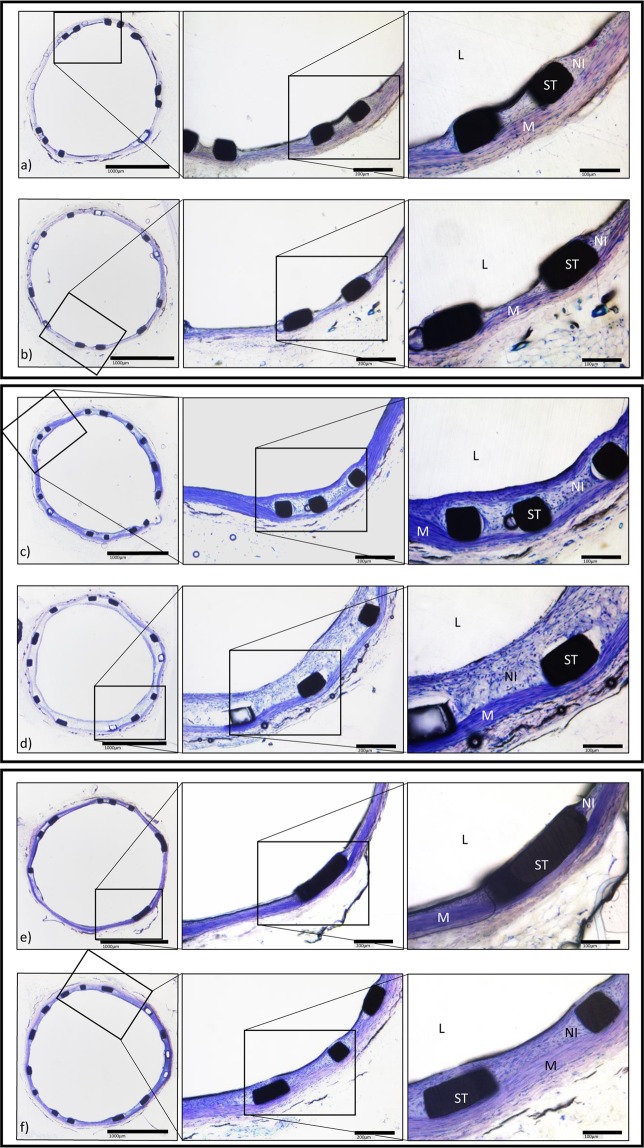
Histology. Representative photomicrographs of Giemsa-stained abdominal aorta at 28 days after stent implantation in wildtype *apoE*^+/+^ rats (upper panel) fed normal diet (**a**) and western diet (**b**), in homozygous *apoE*^−/−^ rats (middle panel) on normal diet (**c**) and western diet (**d**) and on heterozygous *apoE*^+/−^ rats (lower panel) on normal diet (**e**) and western diet. (**f**) (High power images: NI = neointima, St = stent strut, M = tunica media, L = lumen).

Histomorphometric evaluation revealed proliferative neointimal responses and luminal stenosis of varying magnitudes, summarized in Table 2 and Fig. 3. Representative photomicrographs are shown in Fig. 4.

**Table 2:** Histomorphometric measurements after stent implantation in the rat abdominal aorta.

	Wildtype *apoE*^+/+^ rats		Homozygous *apoE*^−/−^ knockout rats		Heterozygous *apoE*^+/−^ knockout rats	
	Normal Diet	Western Diet	Normal Diet	Western Diet	Normal Diet	Western Diet
Restenosis (%)	7.18 ± 0.54		12.44 ± 1.10		7.80 ± 0.79	
	7.44 ± 0.88	7.00 ± 0.68	10.47 ± 0.85	13.72 ± 1.70	6.44 ± 0.98	8.95 ± 1.18
Neointimal area (μm^2^)	245,301 ± 19,545		401,954 ± 36,379		262,804 ± 26,076	
	252,535 ± 31,798	240,040 ± 24,983	315,225 ± 27,842	458,211 ± 55,604	219,885 ± 34,808	229,364 ± 37,257
Neointimal thickness (μm)	38.32 ± 2.98		65.87 ± 5.82		41.45 ± 4.14	
	39.62 ± 4.87	37.37 ± 3.81	53.89 ± 4.63	73.64 ± 8.94	34.36 ± 5.33	47.49 ± 6.01
IEL area (μm^2^)	3,394,754 ± 52,489		3,424,818 ± 92,788		3,475,626 ± 76,789	
	3,333,736 ± 70,893	3,439,131 ± 74,530	3,104,869 ± 78,204	3,632,353 ± 134,468	3,367,053 ± 90,283	3,568,114 ± 118,274
Luminal area (μm^2^)	3,149,453 ± 50,620		3,017,717 ± 94,036		3,212,822 ± 80,523	
	3,081,201 ± 61,964	3,199,091 ± 74,535	2,776,561 ± 70,271	3,174,142 ± 143,262	3,147,168 ± 83,277	3,268,750 ± 131,790

Data are presented as mean ± standard error of the mean.

We assessed in-stent restenosis (ISR) as quotient of stenotic lumen area and original lumen area. As ISR implies substantial neointimal thickening after stent implantation, we assessed the neointimal area as well as the neointimal thickness for each section.

At 28 days, ISR was 7.18 ± 0.54% for wildtype *apoE*^+/+^ rats, 12.44 ± 1.10% for homozygous *apoE*^−/−^, and 7.80 ± 0.79% for heterozygous *apoE*^+/−^ rats. On western diet, restenosis was significantly higher in homozygous *apoE*^−/−^ knockout rats as compared to wildtype *apoE*^+/+^ rats (p = <0.0001) and heterozygous *apoE*^+/−^ rats (p = 0.0102). Similarly, neointimal area was greater in homozygous *apoE*^−/−^ knockout rats than in wildtype *apoE*^+/+^ rats (p = <0.0001) and heterozygous *apoE*^+/−^ rats (p = 0.0103). Furthermore, neointima was significantly thicker in homozygous *apoE*^−/−^ knockout rats as compared to wildtype *apoE*^+/+^ rats (p = <0.0001) and heterozygous *apoE*^+/−^ rats (p = 0.0076) on western diet.

Irrespective of diet or phenotype, elevated TC levels correlated positively with restenosis, whereas neither LDL-C nor HDL-C were significantly associated with restenosis (Fig. 3d–f).

## Discussion

### Atherosclerosis in apolipoprotein E deficient rats

The introduction of nuclease techniques such as Zinc Finger (ZF)^32^, Transcription Activator-Like Effector Nuclease (TALEN) technology^33^, or Clustered Regularly Interspaced Short Palindromic Repeats (CRISPR/Cas9)^34^ enabled the deletion of genes in species other than mice. Since 2011, apolipoprotein E deficient rats are available. These rats develop elevated cholesterol levels and thus mimic human pathophysiology more closely, so the study of ISR and neointimal hyperplasia in *apoE* deficient rats promises an appropriate approach.

So far, only limited data on *apoE* knockout rats have been published, and findings about spontaneous atherosclerosis are contradictory. Wei *et al*. stated that *apoE* knockout rats fed a high fat diet for 12 weeks were resistant to hyperlipidemia-induced atherosclerosis until a partial ligature of the carotid artery was performed^33^. In another study by Rune *et al*., *apoE* knockout rats developed only modest atherosclerotic lesions limited to the aortic sinus after 20 weeks of western diet feeding^36^. By contrast, Zhao *et al*. observed typical atherosclerosis in *apoE* knockout rats after at least 24 weeks on normal or western diet with a continuous increase in plaque burden and lesion severity until sacrifice at 72 weeks^34^. Thus, *apoE* knockout rats were proposed as novel animal model of atherosclerosis promoting also future investigations on intravascular angioplasty and stenting.

For this study, 14 to 16 week old rats underwent aortic stent implantation. Although the *apoE* deficient genotype should make rats more susceptible to atherosclerotic lesions, antecedent atherosclerotic plaques were not found. However, according to the recently published data, it is improbable that rats would develop spontaneous atherosclerosis at 14 to 16 weeks of age. However, restenosis and atherosclerosis are two separate though related pathologic events.

### Impact of genotype on in-stent restenosis, neointimal area and neointimal thickness

*ApoE* deficient mice are more susceptible to both atherosclerotic lesions and ISR. Ali *et al*. reported the first model of ISR in *apoE* deficient mice in 2007^28^. After *ex-vivo* stent implantation into the thoracic aorta and transplantation into the carotid of a syngeneic recipient, at 28 days, neointimal area was 30% greater as compared to wildtype mice. In line with these data, homozygous *apoE*^−/−^ deficient rats exhibited a 39% greater neointimal area and a 42% thicker neointima as compared to wildtype *apoE*^+/+^ rats in our study.

Rodriguez-Menocal *et al*. reported a mean restenosis of 37.96 ± 10.79% 30 days after stent implantation in *apoE* deficient mice^37^. Langeveld *et al*. first introduced the model of abdominal aorta stenting in wildtype *apoE*^+/+^ rats and reported ISR of 18 ± 2.0% after 28 days^29^. Compared to these results, ISR was less pronounced in our study with wildtype *apoE*^+/+^ rats exhibiting an ISR of 7.18 ± 0.54% (7.44 ± 0.88% on normal diet and 7.00 ± 0.68% on western diet). With 12.44 ± 1.1% (10.47 ± 0.85% on normal diet and 13.72 ± 1.70% on western diet), even *apoE*^−/−^ rats did not develop as much ISR as it was reported from Langeveld *et al*. Two major differences might have contributed to a lower extent of ISR in our study. In contrast to Langeveld *et al*., clopidogrel was administered after stent implantation to our rats. As indicated by several research groups, clopidogrel may have anti-inflammatory effects thus diminishing ISR^38,39^. Nevertheless, the administration of clopidogrel appears to be reasonable. As a dual antiplatelet therapy, e.g. clopidogrel, generally is administered to humans after stent implantation, human conditions are mimicked more accurately. Secondly, stent thrombosis occurred in 16% of stented vessels on aspirine therapy only in the *apoE* deficient mouse model^28^.

Furthermore, in contrast to our study, Langeveld used the trans-abdominal and not the trans-iliacal access for stent implantation. This technique, however, requires a physical constriction of the aorta to achieve a temporary interruption of blood flow. The inevitably associated vessel injury and manipulation might potentially cause inflammatory reactions contributing to an increased ISR^31^. Although Oyamada *et al*. did not compare ISR in trans-iliacally vs. trans-abdominally accessed rats in a later study, they reported a lower mortality rate in rats undergoing stent implantation with a trans-iliacal access. Thus, we reasoned to use a trans-iliac access for our experiments. Further studies are needed to evaluate the impact of surgical access on ISR.

Although we observed a generally lower ISR as compared to previous studies, we found homozygous *apoE*^−/−^ rats to exhibit significantly more restenosis, a greater neointimal area and neointimal thickness as compared to wildtype *apoE*^+/+^ rats and heterozygous *apoE*^+/−^ rats on western diet.

Our study demonstrates that homozygous *apoE*^−/−^ knockout rats develop markedly elevated neointimal hyperplasia and in-stent restenosis similar to the well-established *apoE*^−/−^ knockout mice.

### Apolipoprotein E deficiency in rats and mice

In spite of the apparently similar healing process following stent implantation, some important differences between rat and mice arteries have to be emphasized. The endothelial layer in mice arteries lies directly on the internal elastic lamina, and the media consists of only two or three layers of smooth muscle cells^40^. The wall thickness of the aorta ranges between 50μm in young and 85μm in old mice^41^. In rats however, the endothelium is reinforced by sub-endothelial intimal cells and a sub-endothelial space filled with a watery matrix, which is infiltrated by cells, amorphous granulo-vesicular debris and crystalline bodies as the rats age, resulting in a diffuse intimal thickening in old rats^42^. The media consists of multiple layers of densely packed smooth muscle cells and elastic fibers^43^. This translates into different biomechanical and microstructural properties. Due to the thin vessel wall of the mouse aorta, stents have to be deployed using pressure levels as low as 2 atm^37^, which might lead to malapposition of the stent. With an average wall thickness of 146 μm^44^, rat aortas are more resistant towards pressure. For example, we were able to implant the stents with a deployment pressure of 12 atm, a similar deployment pressure as in humans.

### The aorta as target vessel for stent implantation

It remains in part to be established whether the findings in the rat abdominal aorta can be extrapolated to the changes observed in a stented human coronary artery. There are significant differences in hemodynamics, endothelial cell heterogeneity and influences produced by surrounding parenchymal cells between the aorta and coronary arteries.

As the target vessel in the rat, the aorta, is an elastic vessel, some of the vasomotor responses to injury of the human coronary artery, which is a muscular vessel, may not be accurately reproduced. During maturation of the extracellular matrix, Finn *et al*. observed a transition from proteoglycan elements to mostly elastin with some collagen following stent implantation into the carotid artery of the rat^14^. Whereas elastic fibers have been detected in other stent restenosis models, e.g. the rabbit, they are uncommon in human coronary arteries.

Furthermore, the aorta is a large diameter artery with high flow through it, but coronaries are small. Local hemodynamic factors impact neointimal formation. In particular low endothelial shear stress promotes neointimal formation after BMS implantation in human coronary arteries^45,46^. The vascular endothelium of the aorta, however, is exposed to high shear stress conditions that might prevent ISR. Moreover, different vascular beds confer specific susceptibility of the endothelium to different pathologic stimuli^47^.

The deployment of endovascular stents into the human aorta has mostly been associated with pseudoaneurysm formation, stent migration, late stent strut fracture and aortic wall dissection or rupture^48,49^. Thus, the pathologic pattern in aortic stents is different from coronary stents, which are rather affected by stenotic and thrombotic complications.

### Impact of diet on in-stent restenosis, neointimal area and neointimal thickness

Hypercholesterolemia is a well-known risk factor in the development and progression of atherosclerosis and seems to contribute to the development of ISR in humans^50^. Mice and rats are naturally resistant to any dietary intervention-induced increase in cholesterol levels^51^. Mice with an *apoE*^−/−^ background are known to be highly sensitive to dietary interventions exhibiting exceedingly high TC and LDL-C levels^52^ and developing more advanced atherosclerotic lesions on western diet as compared to normal chow^53^.

In our study, TC and LDL-C levels were significantly elevated in homozygous *apoE*^−/−^ rats, on both normal and on western diet. However, in contrast to mice studies, western diet did not further increase TC and LDL-C levels in *apoE*^−/−^ rats after 28 days. As western diet did not start until 3 days before stent implantation, the short diet period of only 4 weeks might explain the lacking additional effect of western diet upon cholesterol levels in homozygous *apoE*^−/−^ rats. In general, homozygous *apoE*^−/−^ rats have been shown to be susceptible to dietary interventions. Rune *et al*. reported markedly elevated TC and LDL-C levels in homozygous *apoE*^−/−^ rats on western diet as compared to normal diet^36^. However, western diet was already fed to the mothers from approximately 10 days pre-partum, and continued to the offspring until 20 weeks of age, underlining the impact of the diet period. In contrast to a homozygous *apoE*^−/−^ background, changes in lipid profile were more pronounced in heterozygous *apoE*^+/−^ rats exhibiting 2.3fold increased TC, 3.3fold increased LDL-C and 3.9fold decreased HDL-C on western diet as compared to normal diet in our study. However, *apoE*^+/−^ rats did not reach TC and LDL-C levels of homozygous *apoE*^−/−^ rats.

A longer period of western diet feeding might be needed to reach a marked elevation of the lipid levels. However, despite the lacking further increase of lipid levels in our study, western diet seemed to promote restenosis in homozygous *apoE*^−/−^ rats. Comparing restenosis, neointimal area and neointimal thickness in wildtype *apoE*^+/+^, homozygous *apoE*^−/−^ and heterozygous *apoE*^+/−^ rats on normal chow, the differences failed to be significant. Western-diet-fed *apoE*^−/−^ rats, however, exhibited significantly more restenosis, a larger neointimal area and a higher neointimal thickness as compared to their western-diet-fed wildtype *apoE*^+/+^ and heterozygous *apoE*^+/−^ littermates. We can only speculate on the reasons for this observation. In mice, deficiency in *apoE* has been shown to render the animals more susceptible to dietary-induced atherosclerosis which might not necessarily reflect in elevated lipid levels: Zhang *et al*. observed a delayed clearance of *apoE* containing particles in heterozygous *apoE*^+/−^ mice on western diet despite normal lipid levels as compared to wildtype *apoE*^+/+^ mice. Whereas western diet did not impact the cholesterol levels in heterozygous *apoE*+/− mice, these animals developed larger lesions than wildtype *apoE*^+/+^ mice^54^. Further studies are needed to provide more in-depth information on the impact of dietary changes upon lipid clearance and how this might lead to an increased susceptibility to atherosclerosis in *apoE* deficient rats.

Western diet feeding has been shown to increase reactive oxygen species^55^. Rune *et al*. observed elevated markers of oxidative stress in *apoE*^−/−^ rats fed western diet as compared to *apoE*^−/−^ rats fed normal chow^36^. Oxidative stress is known to be a critical mediator of inflammation in atherosclerosis^56^ as well as an important molecular mechanism involved in the development of ISR following stent implantation^57^. As we did not investigate reactive oxygen species in our study, this might be an interesting target for further studies about ISR in *apoE*^−/−^ rats with and without western diet feeding.

### Comparison of human in-stent restenosis with apolipoprotein E deficient rats

In our study, homozygous *apoE*^−/−^ rats exhibited a human-like lipoprotein profile with elevated TC and LDL-C levels as well as low HDL-C levels. This is consistent with previous findings on *apoE*^−/−^ rats^33,36^. Hypercholesterolemia has bearing effects on ISR in humans. Patients with high LDL-C and low HDL-C levels are at increased risk for ISR^50,58^. Similar to human data, high TC levels were significantly associated with ISR in our study, and ISR trended to be higher at lower HDL-C levels.

However, the underlying pathophysiologic patterns of ISR are different in humans and rats. In general, the time course of vascular healing after stent implantation is more prolonged in humans than in animals^59^. Peak neointimal growth in bare metal stents (BMS) is observed at 28 days in rats, as compared to 6–12 months in humans^60^. Stent implantation usually is performed in healthy animal arteries without antecedent atherosclerotic plaque lesion, and the developing neointima is more precisely a response to traumatic injury. In contrast, coronary stenting in humans is performed in diseased arteries with at least 70% of the stent directly contacting the underlying atherosclerotic plaque^60^. Due to plaque splitting and medial disruption, stent implantation in humans is often associated with extensive local trauma. Rather than a simple proliferative response, smooth muscle cell migration from within the plaque to the neointima may be the more dominant factor contributing to in-stent restenosis in humans^60^.

The *apoE* deficient genotype should make rats more susceptible to atherosclerotic lesions. However, given that homozygous *apoE*^−/−^ rats did not develop atherosclerotic lesions spontaneously until 24 weeks of age in previous studies^34^, we were unlikely to find antecedent atherosclerosis in our study, as rats were only 14 to 16 weeks of age.

Further approaches might include stent implantation into arterial sites with atherosclerotic lesions induced by prior vascular injury in order to mimic pathophysiology of ISR more appropriately.

### Study limitations

Due to the small size of rats, restenosis and neointimal hyperplasia were evaluated by histomorphometric and not angiographic means as in clinical studies or large animal models. This limitation can result in false positives because absolute changes in histomorphometry may not necessarily be visible in angiography.

For this study, only BMS and no drug-eluting stents (DES) have been implanted to allow pure insights into restenosis in apolipoprotein E deficient rats without the influence of anti-proliferative drugs in DES that might potentially confound the data. The use of DES has overcome the issue of restenosis in clinical practice as restenosis occurs infrequently in drug-eluting stents. Instead of that, the success of DES is limited by pathologies like neoatherosclerosis and late stent thrombosis. To evaluate whether the homozygous *apoE*^−/−^ rat model can mirror all aspects of stent failure beyond restenosis, DES should also be investigated.

## Conclusion

Abdominal aorta stenting of homozygous *apoE*^−/−^ rats is a reliable model to investigate restenosis after stent implantation. Compared to wildtype *apoE*^+/+^ rats and heterozygous *apoE*^+/−^ rats, ISR, neointimal area and neointimal thickness are more pronounced in homozygous *apoE*^−/−^ rats on western diet. Contrary to smaller animal models such as the mouse model, homozygous *apoE*^−/−^ rats enable the evaluation of human-sized coronary stents while at the same time providing an atherogenic phenotype. Further approaches should address the evaluation of drug-eluting stents and other anti-restenotic strategies.

## Materials and Methods

### Animals and animal care

The experimental protocol was approved by the Ministry of Nature, Environment and Country Development (Recklinghausen, Germany; AZ 87-51.04.2010.A065). Animal use respected the guidelines established by the German Council on Animal Care.

Male homozygous *apoE*^−/−^ Sprague-Dawley rats (SD-*apoE*^tm1sage^ rats; product number: TGRA3710HM9; Compor Zr® Zinc-finger nuclease target site: 5′ CAGGCCCTGAACCGAttctggGATTACCTGCGCTGGG; NCBI GeneID: NC_005100.2 GenBank; Sigma Advanced Genetic Engineering Labs, St. Louis, MO) were obtained from SAGE Labs. Male Sprague-Dawley wildtype *apoE*^+/+^ control rats were obtained from Charles River (Sulzfeld, Germany). Heterozygous male *apoE*^+/−^ Sprague Dawley rats were bred in-house. To confirm the genotype of each rat, ear punch tissue from each animal was lysed in QuickExtract DNA Extraction Solution (Biozym, Hessisch Oldendorf, Germany) at 65 °C for 30 minutes followed by heating to 98 °C for 2 minutes. Polymerase chain reaction was set up using REDTaq ReadyMix PCR Reaction Mix (Sigma-Aldrich, St. Louis, MO) with 2.5 μl of each lysate. The genomic region encompassing the zinc-finger nuclease target site was amplified with a specific primer for *apoE*: F, 5′-cgagggagagctggaggt-3′ and R, 5′-tgtgtgacttgggagctctg-3′. The PCR product was loaded directly onto a 3.5% agarose gel. Ready-Load™ DNA Ladder (Invitrogen, Carlsbad, CA) served as molecular weight standard. Wildtype *apoE*^+/+^ bands appeared at 150 bp, and homozygous *apoE*^−/−^ bands could be detected at 134 bp. Heterozygous *apoE*^+/−^ bands were visible at both 150 bp and 134 bp (Supplemental Figs. 1–5).

All animals were kept under identical conditions (21° ± 2 °C, 60% ± 5% humidity, and a 12 hour light/dark cycle). Animals had free access to water containing acetylsalicylic acid (20 mg/l; Aspisol; Bayer Leverkusen, Leverkusen) and food; food was mixed with Clopidogrel (15 mg/kg; Iscover; Bristol-Myers Squibb Pharma, Uxbridge) by Ssniff Spezialdiäten (Soest). A total of 8 wildtype *apoE*^+/+^ rats, 8 homozygous *apoE*^−/−^ and 8 heterozygous *apoE*^+/−^ rats received western diet (WD, Ssniff Spezialdiäten, Soest, Germany), containing 21.3% butter fat and supplemented with 1.25% cholesterol to induce hypercholesterolemia and to enhance plaque formation. Control diet (“normal diet”) contained 3.3% crude fat and was cholesterol-free. Diet was started 3 days before intervention and continued until sacrifice.

### Biochemical analysis

For blood analysis, approximately 1.5 mL was obtained by retro-orbital blood withdrawal and collected in ethylenediaminetetraacetic acid and serum collection tubes (Sarstedt, Nümbrecht). Blood plasma was stored at −80 °C until further analysis. Lipids and lipoproteins including total cholesterol (TC), triglyceride (TG), low-density lipoprotein cholesterol (LDL-C), high-density lipoprotein cholesterol (HDL-C), glucose, creatinine and uric acid levels, as well as blood cell counts (hemogram) were evaluated by the laboratory of the animal facility within the University Hospital Aachen.

### Surgery

Surgery was performed as described earlier^31^. Male wildtype *apoE*^+/+^, homozygous *apoE*^−/−^ and heterozygous *apoE*^+/−^ rats at 14 to 16 weeks of age weighing 440 to 530 g were anesthesized by intra-peritoneal application of ketanest (100 mg/kg; Ceva Santé Animale, Brussels) and xylazin (20 mg/kg; Medistar, Ascheberg). Surgery was conducted using a dissecting microscope (Leica MZ9, Wetzlar). Stent implantation was performed by two experienced surgeons using trans-iliacal access in all 42 rats. An incision hole was made in the left iliac artery to introduce the stent (Multilink MiniVision 2.5 × 8 mm; Abbott Vascular, St. Clara, CA). Multilink MiniVision is a thin strut cobalt-chromium coronary stent that has been well investigated in different settings in multiple study registries as well as randomized controlled trials^35^.

The vessel was flushed with heparin in 0.9% NaCl (200IU/mL) and the catheter was passed along the vessel in the direction of the aortic arch. The balloon was inflated to 12 atm at a suprarenal abdominal level with a Medflator (Medex Supply; Monsey, NY) for 15 sec. After removal of the balloon, the arterial incision was closed using a 9-0 non-degradable suture (Dafilon; Aesculap, Tuttlingen). The skin incision was closed using 4-0 permanent sutures (Silkam; Aesculap, Tuttlingen). Analgesia was maintained by 0.01–0.05 mg/kg Buprenorphin (Temgesic; Essex Pharma, Munich) for 72 h. Overstretch ratio (ratio between stented vessel and native vessel diameter) in all stented vessels was 1.2:1.

### Protocol

The study protocol is summarized in Fig. 1. A total of 42 rats (16 wildtype *apoE*^+/+^, 13 *apoE*^−/−^ and 13 *apoE*^+/−^ rats) underwent surgery and stent implantation. Among the wildtype *apoE*^+/+^ rats, 8 animals received normal diet and 8 animals received western diet. In each group of homozygous *apoE*^−/−^ and heterozygous *apoE*^+/−^ rats, 6 animals were fed normal and 7 animals were fed western diet, respectively. During surgery, 1 wildtype *apoE*^+/+^ and 1 heterozygous *apoE*^+/−^ rat had to be sacrificed immediately after stent deployment due to vessel closure failure. 1 wildtype *apoE*^+/+^ and 1 homozygous *apoE*^−/−^ rat were symptomatic for stent thrombosis and thus sacrificed within 24 hours. 1 wildtype *apoE*^+/+^ and 1 heterozygous *apoE*^+/−^ rat died prematurely from internal hemorrhage between 5 and 17 days post intervention.

A total of 13 wildtype *apoE*^+/+^, 12 homozygous *apoE*^−/−^ and 11 heterozygous *apoE*^+/−^ rats received successful aortic stent implantation. The stented aortas of 1 wildtype *apoE*^+/+^, 1 homozygous *apoE*^−/−^ and 1 heterozygous *apoE*^+/−^ rat were not analyzable due to processing failure. Thus, we analyzed ISR, neointimal area and neointimal thickness in 12 wildtype *apoE*^+/+^ (5 on normal diet, 7 on western diet), 11 homozygous *apoE*^−/−^ (4 on normal diet and 7 on western diet) and 10 heterozygous *apoE*^+/−^ rats (5 on normal diet and 5 on western diet).

### Histopathologic analysis

After fixation with 4% paraformaldehyde for 24 h, tissue samples were processed by embedding in methylmethacrylate (KPlast; Medizinische Diagnostik-Methoden, Giessen). Sections of 10- to 12-μm thickness were cut from the proximal, middle and distal parts of the stents via a sawing-and-grinding technique. Each section was polished and analyzed using routine histochemical staining. In all sections, histological analysis was performed by an independent observer.

### Histomorphometric analysis

Histomorphometric analysis was performed of sequential sections of the proximal, middle and distal part of the entire stent length using digital morphometry by means of a Leica DMI-3000 microscope (Wetzlar) and DISKUS image analysis software (version 4.80, C. Hilgers Technical Bureau, Königswinter). The internal elastic lamina (IEL) as well as the lumen were traced with a Tavla Pen Tablet (Braun Photo Technik, Nuremberg). From these values, IEL area and lumen area were calculated by the software.

For each section, percent cross-sectional area in-stent restenosis was calculated as:

$${\rm{Restenosis}}\,( \% )=100\times (1-[{\rm{lumen}}\,{\rm{area}}/{\rm{IEL}}\,{\rm{area}}])$$

Total neointimal area (A_i_) was calculated as the difference between the area inside the internal elastic lamina (IEL) and the lumen area^61^:

$${{\rm{A}}}_{{\rm{i}}}=({\rm{IEL}}\,{\rm{area}}-{\rm{lumen}}\,{\rm{area}})$$

Neointimal thickness (NIT) was calculated as:

$${\rm{NIT}}=(2\times {{\rm{A}}}_{{\rm{i}}})/({{\rm{P}}}_{{\rm{L}}}+{{\rm{P}}}_{{\rm{IEL}}})$$

where P_L_ and P_IEL_ are the lumen and internal elastic lamina perimeter, respectively^62^.

### Statistical analysis

All results are expressed as mean ± SEM. Statistical significance was evaluated by testing of multiple comparisons by analysis of variance. When the analysis of variance demonstrated significant differences, individual mean differences were analyzed by Tukey’s multiple comparison test. Unpaired two-tailed t test was used for comparing means of two groups. For all statistical analyses, p < 0.05 was considered significant. For statistical analysis, GraphPad Prism (version 7.04, GraphPad Software, Inc., San Diego, CA) was used.

## Supplementary information

Supplementary Material

## Data Availability

The datasets generated during and/or analyzed during the current study are available from the corresponding author on reasonable request.

## References

[1] Patel MR (2017). ACC/AATS/AHA/ASE/ASNC/SCAI/SCCT/STS 2017 Appropriate Use Criteria for Coronary Revascularization in Patients With Stable Ischemic Heart Disease: A Report of the American College of Cardiology Appropriate Use Criteria Task Force, American Association for Thoracic Surgery, American Heart Association, American Society of Echocardiography, American Society of Nuclear Cardiology, Society for Cardiovascular Angiography and Interventions, Society of Cardiovascular Computed Tomography, and Society of Thoracic Surgeons. Journal of the American College of Cardiology.

[2] Virmani R, Farb A (1999). Pathology of in-stent restenosis. Curr Opin Lipidol.

[3] Schwartz RS, Chronos NA, Virmani R (2004). Preclinical restenosis models and drug-eluting stents: still important, still much to learn. Journal of the American College of Cardiology.

[4] Gravanis MB, Roubin GS (1989). Histopathologic phenomena at the site of percutaneous transluminal coronary angioplasty: the problem of restenosis. Human pathology.

[5] Pasternak RC, Baughman KL, Fallon JT, Block PC (1980). Scanning electron microscopy after coronary transluminal angioplasty of normal canine coronary arteries. The American journal of cardiology.

[6] Faxon DP (1982). Acute effects of transluminal angioplasty in three experimental models of atherosclerosis. Arteriosclerosis.

[7] Davies PF (2009). Hemodynamic shear stress and the endothelium in cardiovascular pathophysiology. Nat Clin Pract Cardiovasc Med.

[8] Wilentz JR (1987). Platelet accumulation in experimental angioplasty: time course and relation to vascular injury. Circulation.

[9] Liu MW, Roubin GS, King SB (1989). Restenosis after coronary angioplasty. Potential biologic determinants and role of intimal hyperplasia. Circulation.

[10] Phillips-Hughes J, Kandarpa K (1996). Restenosis: pathophysiology and preventive strategies. J Vasc Interv Radiol.

[11] Thyberg J, Hedin U, Sjolund M, Palmberg L, Bottger BA (1990). Regulation of differentiated properties and proliferation of arterial smooth muscle cells. Arteriosclerosis.

[12] Austin GE, Ratliff NB, Hollman J, Tabei S, Phillips DF (1985). Intimal proliferation of smooth muscle cells as an explanation for recurrent coronary artery stenosis after percutaneous transluminal coronary angioplasty. Journal of the American College of Cardiology.

[13] Waller BF (1991). Morphological observations late (greater than 30 days) after clinically successful coronary balloon angioplasty. Circulation.

[14] Finn AV (2002). A novel rat model of carotid artery stenting for the understanding of restenosis in metabolic diseases. J Vasc Res.

[15] Touchard AG, Schwartz RS (2006). Preclinical restenosis models: challenges and successes. Toxicol Pathol.

[16] Perkins LE (2010). Preclinical models of restenosis and their application in the evaluation of drug-eluting stent systems. Vet Pathol.

[17] Kim WH (2000). Histopathologic analysis of in-stent neointimal regression in a porcine coronary model. Coron Artery Dis.

[18] Watanabe Y (1980). Serial inbreeding of rabbits with hereditary hyperlipidemia (WHHL-rabbit). Atherosclerosis.

[19] Atkinson JB, Hoover RL, Berry KK, Swift LL (1989). Cholesterol-fed heterozygous Watanabe heritable hyperlipidemic rabbits: a new model for atherosclerosis. Atherosclerosis.

[20] Breslow JL (1993). Transgenic mouse models of lipoprotein metabolism and atherosclerosis. Proc Natl Acad Sci USA.

[21] Knowles JW, Maeda N (2000). Genetic modifiers of atherosclerosis in mice. Arteriosclerosis, thrombosis, and vascular biology.

[22] de Winther MP, Hofker MH (2002). New mouse models for lipoprotein metabolism and atherosclerosis. Curr Opin Lipidol.

[23] Mahley RW (1988). Apolipoprotein E: cholesterol transport protein with expanding role in cell biology. Science.

[24] Meir KS, Leitersdorf E (2004). Atherosclerosis in the apolipoprotein-E-deficient mouse: a decade of progress. Arteriosclerosis, thrombosis, and vascular biology.

[25] Ali K, Middleton M, Pure E, Rader DJ (2005). Apolipoprotein E suppresses the type I inflammatory response *in vivo*. Circulation research.

[26] Piedrahita JA, Zhang SH, Hagaman JR, Oliver PM, Maeda N (1992). Generation of mice carrying a mutant apolipoprotein E gene inactivated by gene targeting in embryonic stem cells. Proc Natl Acad Sci USA.

[27] Plump AS (1992). Severe hypercholesterolemia and atherosclerosis in apolipoprotein E-deficient mice created by homologous recombination in ES cells. Cell.

[28] Ali ZA (2007). Increased in-stent stenosis in *ApoE* knockout mice: insights from a novel mouse model of balloon angioplasty and stenting. Arteriosclerosis, thrombosis, and vascular biology.

[29] Langeveld B (2004). Rat abdominal aorta stenting: a new and reliable small animal model for in-stent restenosis. J Vasc Res.

[30] Indolfi C (2000). A new rat model of small vessel stenting. Basic Res Cardiol.

[31] Oyamada S (2011). Trans-iliac rat aorta stenting: a novel high throughput preclinical stent model for restenosis and thrombosis. J Surg Res.

[32] Ekuni D (2014). Occlusal disharmony accelerates the initiation of atherosclerosis in *apoE* knockout rats. Lipids Health Dis.

[33] Wei S (2015). Apolipoprotein E-deficient rats develop atherosclerotic plaques in partially ligated carotid arteries. Atherosclerosis.

[34] Zhao Y (2018). Hyperlipidemia induces typical atherosclerosis development in Ldlr and *Apoe* deficient rats. Atherosclerosis.

[35] Testa L (2011). Multi-Link Vision stent vs. first-generation drug-eluting stents: systematic review and meta-analysis. Qjm.

[36] Rune I (2018). Long-term Western diet fed apolipoprotein E-deficient rats exhibit only modest early atherosclerotic characteristics. Sci Rep.

[37] Rodriguez-Menocal L (2010). A novel mouse model of in-stent restenosis. Atherosclerosis.

[38] Li M, Zhang Y, Ren H, Zhu X (2007). Effect of clopidogrel on the inflammatory progression of early atherosclerosis in rabbits model. Atherosclerosis.

[39] Herbert JM, Tissinier A, Defreyn G, Maffrand JP (1993). Inhibitory effect of clopidogrel on platelet adhesion and intimal proliferation after arterial injury in rabbits. Arteriosclerosis and thrombosis: a journal of vascular biology.

[40] Xu Q (2004). Mouse models of arteriosclerosis: from arterial injuries to vascular grafts. Am J Pathol.

[41] Wheeler JB, Mukherjee R, Stroud RE, Jones JA, Ikonomidis JS (2015). Relation of murine thoracic aortic structural and cellular changes with aging to passive and active mechanical properties. J Am Heart Assoc.

[42] Gerrity RG, Cliff WJ (1972). The aortic tunica intima in young and aging rats. Experimental and molecular pathology.

[43] Briones AM, Salaices M, Vila E (2007). Mechanisms underlying hypertrophic remodeling and increased stiffness of mesenteric resistance arteries from aged rats. J Gerontol A Biol Sci Med Sci.

[44] Shou Y, Jan KM, Rumschitzki DS (2006). Transport in rat vessel walls. I. Hydraulic conductivities of the aorta, pulmonary artery, and inferior vena cava with intact and denuded endothelia. Am J Physiol Heart Circ Physiol.

[45] Koskinas KC, Chatzizisis YS, Antoniadis AP, Giannoglou GD (2012). Role of endothelial shear stress in stent restenosis and thrombosis: pathophysiologic mechanisms and implications for clinical translation. Journal of the American College of Cardiology.

[46] Papafaklis MI (2010). The effect of shear stress on neointimal response following sirolimus- and paclitaxel-eluting stent implantation compared with bare-metal stents in humans. JACC. Cardiovascular interventions.

[47] Aird W. C. (2011). Endothelial Cell Heterogeneity. Cold Spring Harbor Perspectives in Medicine.

[48] Harrison DA, McLaughlin PR, Lazzam C, Connelly M, Benson LN (2001). Endovascular stents in the management of coarctation of the aorta in the adolescent and adult: one year follow up. Heart.

[49] Lu WH (2017). Clinical Impact of Stent Implantation for Coarctation of the Aorta with Associated Hypoplasia of the Transverse Aortic Arch. Pediatr Cardiol.

[50] Cai A (2013). Baseline LDL-C and Lp(a) elevations portend a high risk of coronary revascularization in patients after stent placement. Dis Markers.

[51] Bobkova D, Honsova E, Kovar J, Poledne R (2004). Effect of diets on lipoprotein concentrations in heterozygous apolipoprotein E-deficient mice. Physiol Res.

[52] van Ree JH (1994). Diet-induced hypercholesterolemia and atherosclerosis in heterozygous apolipoprotein E-deficient mice. Atherosclerosis.

[53] Nakashima Y, Plump AS, Raines EW, Breslow JL, Ross R (1994). *ApoE*-deficient mice develop lesions of all phases of atherosclerosis throughout the arterial tree. Arteriosclerosis and thrombosis: a journal of vascular biology.

[54] Zhang SH, Reddick RL, Burkey B, Maeda N (1994). Diet-induced atherosclerosis in mice heterozygous and homozygous for apolipoprotein E gene disruption. The Journal of clinical investigation.

[55] Miller, V. M., Aarhus, L. L. & Vanhoutte, P. M. Modulation of endothelium-dependent responses by chronic alterations of blood flow. *Am J Physiol* **251** (1986). 10.1152/ajpheart.1986.251.3.H520 3752266

[56] Papac-Milicevic N, Busch CJ, Binder CJ (2016). Malondialdehyde Epitopes as Targets of Immunity and the Implications for Atherosclerosis. Adv Immunol.

[57] Gallo G (2018). Role of oxidative stress in the process of vascular remodeling following coronary revascularization. Int J Cardiol.

[58] Ucar FM (2016). A potential marker of bare metal stent restenosis: monocyte count - to- HDL cholesterol ratio. BMC Cardiovasc Disord.

[59] Farb A (1999). Pathology of acute and chronic coronary stenting in humans. Circulation.

[60] Virmani R, Kolodgie FD, Farb A, Lafont A (2003). Drug eluting stents: are human and animal studies comparable?. Heart.

[61] Vogt F (2004). Long-term assessment of a novel biodegradable paclitaxel-eluting coronary polylactide stent. European heart journal.

[62] Jiang Z (2004). A novel vein graft model: adaptation to differential flow environments. Am J Physiol Heart Circ Physiol.

