# Supplementary material for: Apolipoprotein E deficient rats generated via zinc-finger nucleases exhibit pronounced in-stent restenosis
Source: Sci Rep. 2019 Dec 3;9:18153. doi: 10.1038/s41598-019-54541-z (PMC6890749; doi:10.1038/s41598-019-54541-z)
Supplement: Supplementary file 1 — Supplementary Material [file 41598_2019_54541_MOESM1_ESM.pdf]

Supplementary Material

Apolipoprotein E deficient rats generated via zinc-finger nucleases exhibit pronounced in-stent restenosis

Anne Cornelissen, MD, Sakine Simsekyilmaz, PhD, Elisa Liehn, MD, Mihaela Rusu, BS, Nicole Schaaps, BS, Mamdouh Afify, MD, Roberta Florescu, MD, Mohammad Almalla, MD, Mauricio Borinski, PhD, Felix Vogt, MD

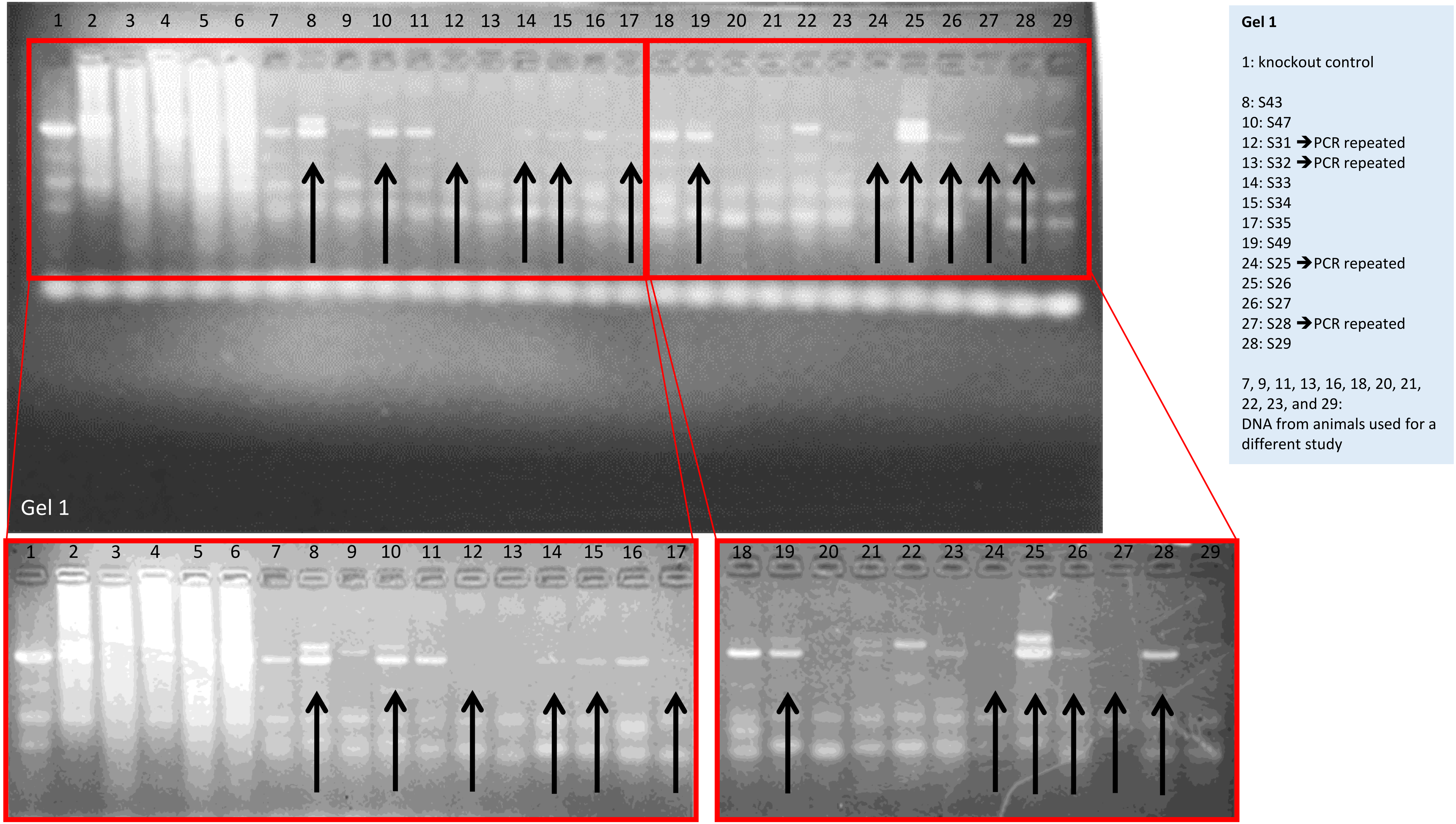

Supplemental Figure 1: Genotyping of rats with polymerase chain reaction using a specific primer for *apoE*. Figure depicts gel no. 1. Wildtype *apoE*$^{+/+}$ bands appear at 150 bp, homozygous *apoE*$^{-/-}$ bands can be detected at 134 bp. Heterozygous *apoE*$^{+/-}$ bands are visible at both 150 bp and 134 bp.

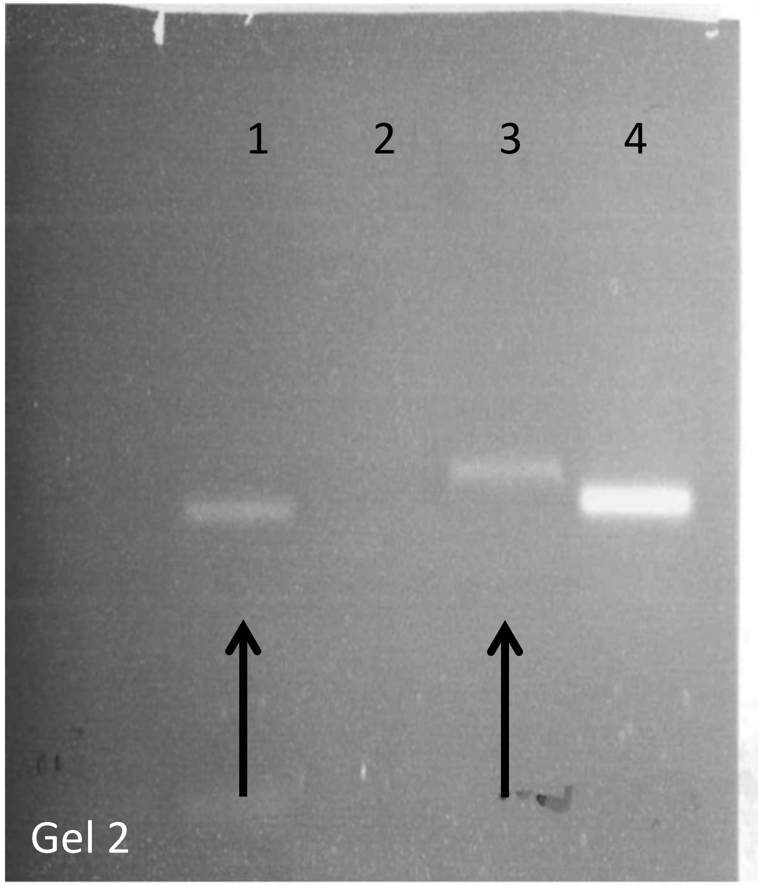

Gel 2

1: repeated PCR S32
3: repeated PCR S28
4: knockout control

2: DNA from animal used for a different study

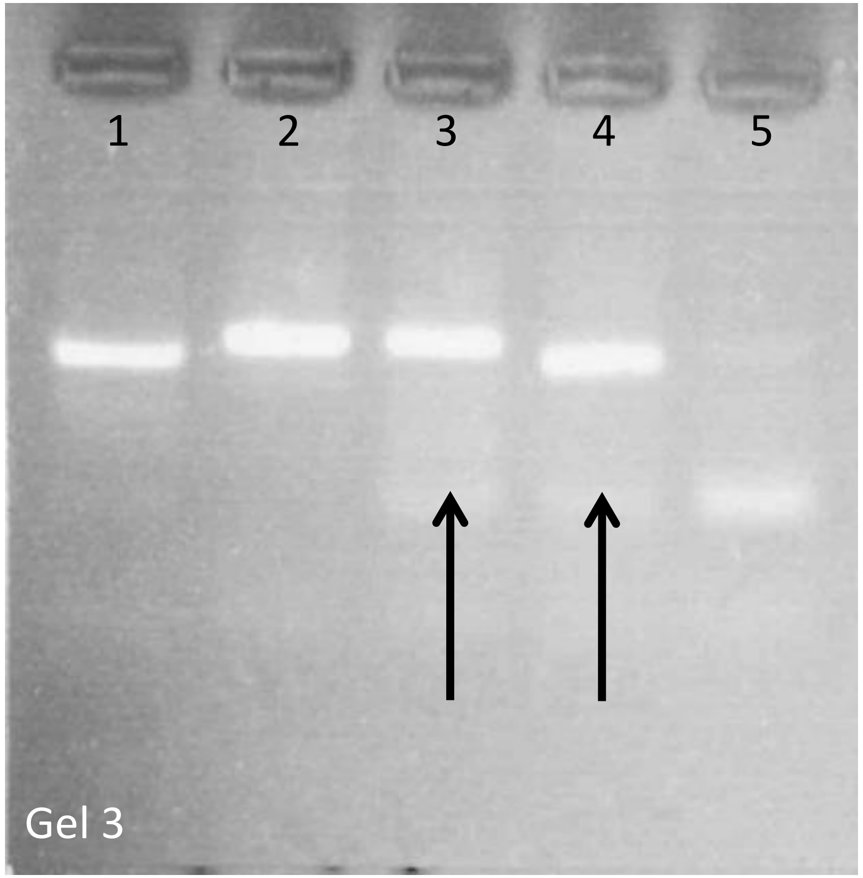

Gel 3

1: knockout control
3: repeated PCR S31
4: repeated PCR S25

2, 5: DNA from animals used for a different study

Supplemental Figure 2: Repeated PCR of failed PCR genotyping of rats using a specific primer for apolipoprotein E. Figure depicts gels no. 2 and 3.

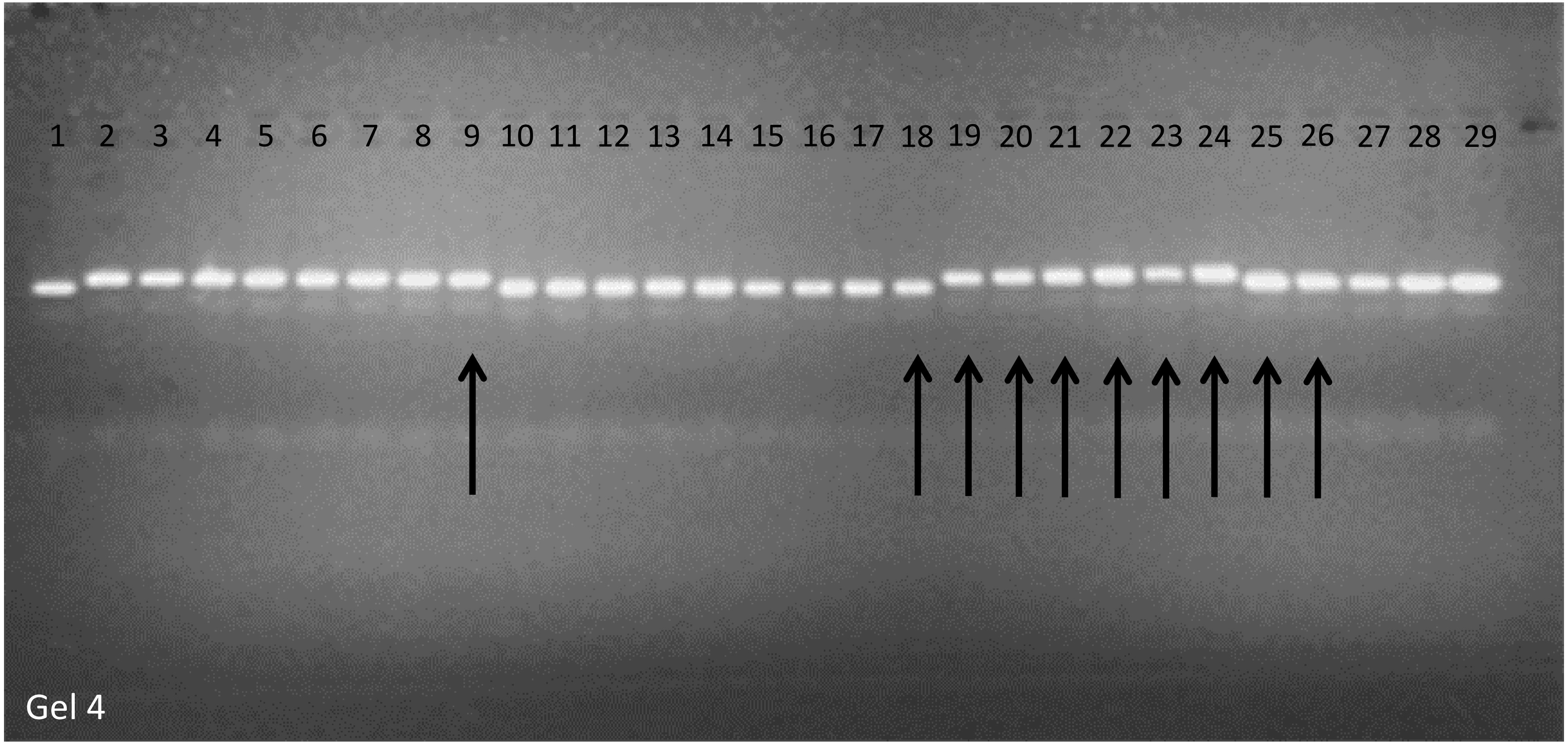

Gel 4

1: knockout control
9: S45
18: S36
19: S37
20: S40
21: S44
22: S51
23: S53
24: S63
25: S61
26: S62
29: knockout control

2, 3, 4, 5, 6, 7, 8, 10, 11, 12, 13, 14, 15, 16, 17, 27, 28: DNA from animals used for a different study

Supplemental Figure 3: Genotyping of rats with polymerase chain reaction using a specific primer for apoE. Figure depicts gel no. 4. Wildtype apo$E^{+/+}$ bands appear at 150 bp, homozygous apo$E^{-/-}$ bands can be detected at 134 bp. Heterozygous apo$E^{+/-}$ bands are visible at both 150 bp and 134 bp.

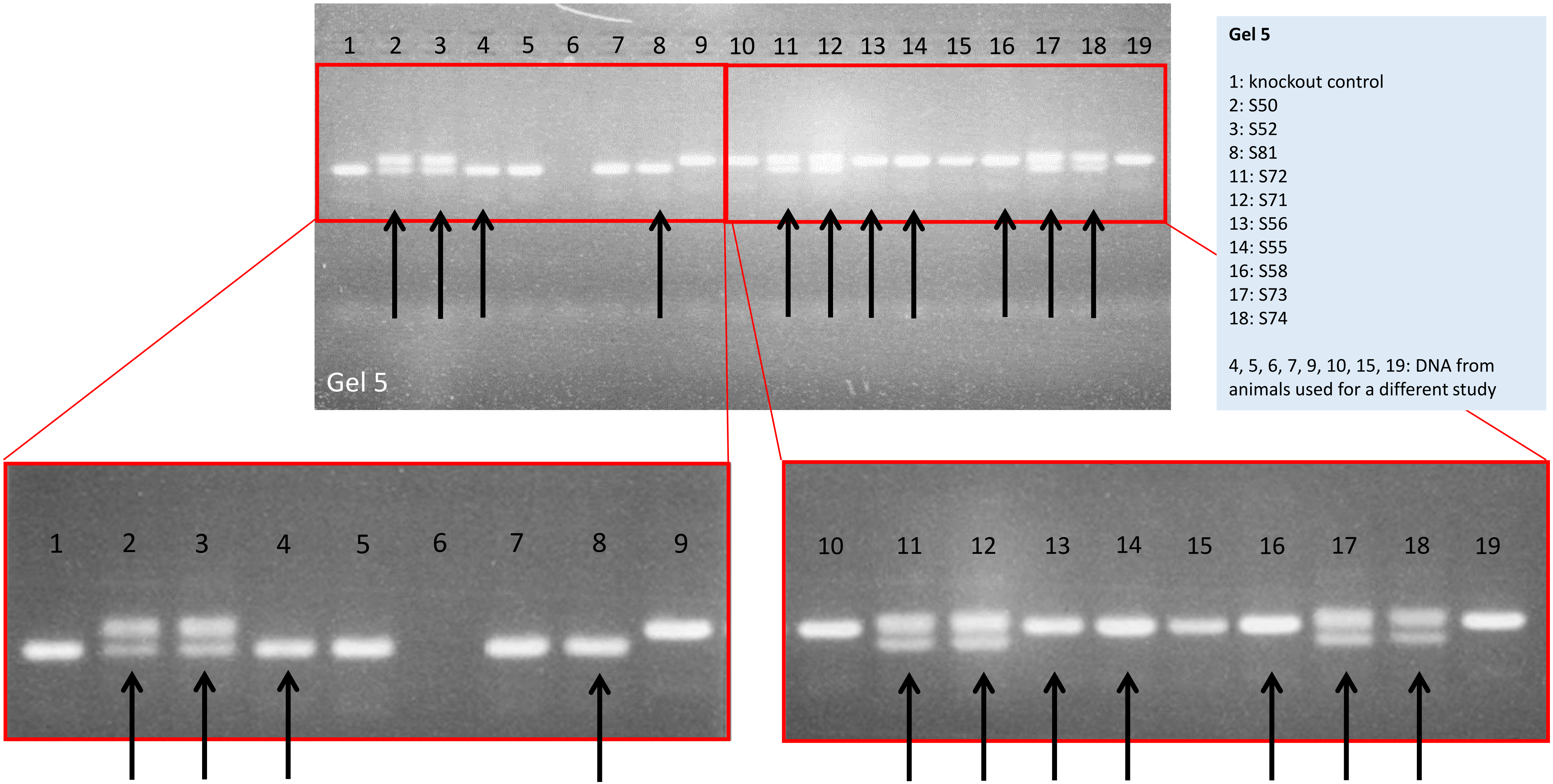

Supplemental Figure 4: Genotyping of rats with polymerase chain reaction using a specific primer for apoE. Figure depicts gel No. 5. Wildtype $apoE^{+/+}$ bands appear at 150 bp, homozygous $apoE^{-/-}$ bands can be detected at 134 bp. Heterozygous $apoE^{+/-}$ bands are visible at both 150 bp and 134 bp.

Animal Number	Gel No.	*ApoE* Genotype
S37	4, Lane 19	+/+
S44	4, Lane 21	+/+
S45	4, Lane 9	+/+
S55	5, Lane 14	+/+
S56	5, Lane 13	+/+
S28	1, Lane 27; repeated on Gel 2, Lane 3	+/+
S31	1, Lane 12; repeated on Gel 3, Lane 3	+/+
S40	4, Lane 20	+/+
S51	4, Lane 22	+/+
S53	4, Lane 23	+/+
S58	5, Lane 16	+/+
S63	4, Lane 24	+/+
S36	4, Lane 18	-/-
S61	4, Lane 25	-/-
S62	4, Lane 26	-/-
S81	5, Lane 8	-/-
S25	1, Lane 24; repeated on Gel 3, Lane 4	-/-
S27	1, Lane 26	-/-
S29	1, Lane 28	-/-
S32	1, Lane 13; repeated on Gel 2, Lane 1	-/-
S33	1, Lane 14	-/-
S34	1, Lane 15	-/-
S35	1, Lane 17	-/-
S43	1, Lane 8	+/-
S47	1, Lane 10	+/-
S49	1, Lane 19	+/-
S73	5, Lane 17	+/-
S74	5, Lane 18	+/-
S26	1, Lane 25	+/-
S50	5, Lane 2	+/-
S52	5, Lane 3	+/-
S71	5, Lane 12	+/-
S72	5, Lane 11	+/-

Supplemental Figure 5: Summary of genotyping results
